# A Target-Based High Throughput Screen Yields *Trypanosoma brucei* Hexokinase Small Molecule Inhibitors with Antiparasitic Activity

**DOI:** 10.1371/journal.pntd.0000659

**Published:** 2010-04-13

**Authors:** Elizabeth R. Sharlow, Todd A. Lyda, Heidi C. Dodson, Gabriela Mustata, Meredith T. Morris, Stephanie S. Leimgruber, Kuo-Hsiung Lee, Yoshiki Kashiwada, David Close, John S. Lazo, James C. Morris

**Affiliations:** 1 University of Pittsburgh Drug Discovery Institute and Pittsburgh Molecular Libraries Screening Center, University of Pittsburgh, Pittsburgh, Pennsylvania, United States of America; 2 Department of Pharmacology and Chemical Biology, University of Pittsburgh, Pittsburgh, Pennsylvania, United States of America; 3 Department of Genetics and Biochemistry, Clemson University, Clemson, South Carolina, United States of America; 4 Department of Computational Biology, University of Pittsburgh, Pittsburgh, Pennsylvania, United States of America; 5 Natural Products Research Laboratories, UNC Eshelman School of Pharmacy, University of North Carolina, Chapel Hill, North Carolina, United States of America; 6 Graduate School of Pharmaceutical Sciences, University of Tokushima, Tokushima, Japan; Swiss Tropical Institute, Switzerland

## Abstract

**Background:**

The parasitic protozoan *Trypanosoma brucei* utilizes glycolysis exclusively for ATP production during infection of the mammalian host. The first step in this metabolic pathway is mediated by hexokinase (TbHK), an enzyme essential to the parasite that transfers the γ-phospho of ATP to a hexose. Here we describe the identification and confirmation of novel small molecule inhibitors of bacterially expressed TbHK1, one of two TbHKs expressed by *T. brucei*, using a high throughput screening assay.

**Methodology/Principal Findings:**

Exploiting optimized high throughput screening assay procedures, we interrogated 220,233 unique compounds and identified 239 active compounds from which ten small molecules were further characterized. Computation chemical cluster analyses indicated that six compounds were structurally related while the remaining four compounds were classified as unrelated or singletons. All ten compounds were ∼20-17,000-fold more potent than lonidamine, a previously identified TbHK1 inhibitor. Seven compounds inhibited *T. brucei* blood stage form parasite growth (0.03≤EC_50_<3 µM) with parasite specificity of the compounds being demonstrated using insect stage *T. brucei* parasites, *Leishmania* promastigotes, and mammalian cell lines. Analysis of two structurally related compounds, ebselen and SID 17387000, revealed that both were mixed inhibitors of TbHK1 with respect to ATP. Additionally, both compounds inhibited parasite lysate-derived HK activity. None of the compounds displayed structural similarity to known hexokinase inhibitors or human African trypanosomiasis therapeutics.

**Conclusions/Significance:**

The novel chemotypes identified here could represent leads for future therapeutic development against the African trypanosome.

## Author Summary

African sleeping sickness is a disease found in sub-Saharan Africa that is caused by the single-celled parasite *Trypanosoma brucei*. The drugs used widely now to treat infections are 50 years old and notable for their toxicity, emphasizing the need for development of new therapeutics. In the search for potential drug targets, researchers typically focus on enzymes or proteins that are essential to the survival of the infectious agent while being distinct enough from the host to avoid accidental targeting of the host enzyme. This work describes our research on one such trypanosome enzyme, hexokinase, which is a protein that the parasite requires to make energy. Here we describe the results of our search for inhibitors of the parasite enzyme. By screening 220,223 compounds for anti-hexokinase activity, we have identified new inhibitors of the parasite enzyme. Some of these are toxic to trypanosomes while having no effect on mammalian cells, suggesting that they may hold promise for the development of new anti-parasitic compounds.

## Introduction

African sleeping sickness conjures historical images of disease-induced fatal slumbering striking down men, women, and children, consequently decimating villages of colonial Africa. Unfortunately, people living in many countries of sub-Saharan Africa today know that African sleeping sickness is *not* a disease of history but rather is a much-neglected disease of the present, particularly in areas that suffer the additional burdens of war, famine, global and local climate changes, and other infectious agents. The causative agents of sleeping sickness (or human African trypanosomiasis, HAT) are subspecies of the African trypanosome *Trypanosoma brucei*. Approximately 500,000 people in sub-Saharan Africa are infected annually with the parasite leading to 50,000–70,000 deaths per year [1]. Similar to other neglected tropical diseases, limited therapeutics for HAT are available and of the drugs currently used, most have serious adverse side effects, including encephalopathy, toxicity, and death [2]. Thus, there is a desperate need for new HAT therapeutics with the preference shifting from general cytotoxic agents towards molecular target-based therapeutics that should display fewer toxic effects.

Bloodstream form (BSF) *T. brucei* parasites generate ATP exclusively through glycolysis and *T. brucei* hexokinase TbHK, the first enzyme in glycolysis, has previously been validated as a target for therapeutic development. In these experiments, BSF parasites were shown to be sensitive to RNA interference (RNAi)-based silencing of TbHKs [3], [4], with cell toxicity observed after 3–5 days of RNAi exposure. Additonally, known inhibitors of HKs have been demonstrated to inhibit *T. brucei* hexokinase 1 (TbHK1), one of two nearly identical TbHKs that the parasite expresses. These compounds are furthermore toxic to the parasite [4]. While some mammalian HK inhibitors can inhibit TbHK1, TbHK1 is distinct enough from mammalian HKs to suggest that it can be specifically targeted. Supporting this notion, TbHK1 shares only 30–33% sequence identity with the mammalian HKs and differs further by unusual oligomerization into hexamers [5]. Moreover, the unusual spectrum of known inhibitors of the trypanosome enzymes, including fatty acids and other small molecules (like pyrophosphate, [5]), support the idea that this essential parasite protein is sufficiently distinct from any mammalian counterpart to make an ideal target for therapeutic development. Indeed, targeting TbHK using structurally based inhibitors has yielded trypanocidal compounds, albeit at high concentrations [6], [7].

Here we describe our high throughput target-based approach to identify specific inhibitors of the essential parasite enzyme, TbHK1. Overall, ten compounds were confirmed as novel TbHK1 small molecule inhibitors exhibiting little or no similarity to known HK inhibitors (or HAT therapeutics). Most of the potent TbHK1 inhibitors were toxic to culture-grown BSF *T. brucei* while not exhibiting toxicity towards mammalian cells, suggesting that they may be useful lead compounds in the development of new therapies for African trypanosomiasis.

## Methods

### Chemicals and reagents

Clear 384-well microtiter plates were purchased from Greiner (Monroe, NC) and used for all experiments. Glucose-6-phosphate dehydrogenase, β-nicotinamide adenine dinucleotide (NAD^+^), adenosine triphosphate (ATP), lipoic acid (PubChem SID 11532893) and glucose were purchased from Sigma (St. Louis, MO). Phosphoenol pyruvate (PEP), ebselen (PubChem SID 856002) and glucosamine were obtained through VWR (West Chester, PA) and dimethyl sulfoxide (DMSO) was purchased from Fisher (Pittsburgh, PA). The following PubChem SID compounds were obtained from commercial vendors: 3716597, 24830882, 17386310, and 16952891 (Enamine/Kiev, Ukraine); 24797131 (Chembridge/San Diego, CA); 14728414 and 17387000 (Specs/Delft, The Netherlands); 17507245 (Asinex/Moscow, Russia); and 24785302 (ChemDiv, San Diego, CA).

### Compound libraries

The library of pharmacologically active compounds (LOPAC) (1,280 compounds) was purchased from Sigma-Aldrich. The Pittsburgh Molecular Libraries Screening Center (PMLSC) provided the 220,233 compound library screened for TbHK1 small molecule inhibitors, which was made available as part of the NIH Molecular Libraries Roadmap Initiative. Cherry-picked compounds from the PMLSC library were supplied by BiofocusDPI (San Francisco, CA).

### Purification of bacterially expressed TbHK1

For purification of bacterially expressed TbHK1 (rTbHK1), a previously described protocol [8] was modified to increase yield. Briefly, a starter culture of *E. coli* M15(pREP) harboring pQE30 (Qiagen, Valencia, CA) with the TbHK1 gene cloned in frame of a 6-His tagging sequence was grown in ECPM1 [9] and then inoculated into a 5 L bioreactor (Biostat B, B. Braun Biotech International, Allentown, PA) and grown at 37°C. At OD_600_ between 3–5, the culture was induced with IPTG (0.8 mM), grown without supplement O_2_ (37°C, 16 hr), and cells collected by centrifugation (5000×g, 20 min, 4°C). The pellet was resuspended in lysis buffer (50 mM NaPO_4_, pH 8.1, 5 mM glucose, 150 mM NaCl, and 0.1% Tween) and lysed by using a cell disruptor (Constant Cell Disruption Systems, Sanford, NC). The resulting supernatant was applied (5 ml/min) to a 50 ml ProBind column (Invitrogen, Eugene, OR) on a FPLC (GE Lifesciences, Piscataway, NJ) and protein eluted by gradient (5 to 250 mM imidazole) in lysis buffer. Fractions were screened using HK activity assays and Western blotting and those containing rTbHK1 were pooled, concentrated, and applied to a HiTrap SP HP column (GE Lifesciences, Piscataway, NJ).

### Automated primary TbHK1 HTS and glucose-6-phosphate dehydrogenase coupled assays

TbHK1 assays were an adaptation of a coupled enzyme HK assay to a 384-well format [8], [10]. Briefly, test and control compounds (30 *µ*M in 15 *µ*L volume) were added to a 384 well black, opaque microtiter plate using a Velocity 11 V-prep (Santa Clara, CA) for a final test compound concentration of 10 µM. Negative (vehicle) controls contained 1% DMSO, positive controls contained 133 mM glucosamine and IC_50_ controls contained 1.3 mM glucosamine (final well concentrations). A mixture containing glucose (1.5 mM), ATP (1.05 mM), MgCl_2_ (4.5 mM), NAD^+^ (9 mM), glucose-6-phosphate dehydrogenase (G6PDH, 0.018 mUnits/µL) and triethanolamine (TEA, 100 mM, pH 8.0) in a 15 µL volume was then added to each well of the assay plate using a Perkin Elmer FlexDrop (Waltham, MA) followed by addition of rTbHK1 (1.5 ng/µl in 15 µL volume). The 45 µL reaction mixture was incubated at RT for 2 hr and then quenched with 5 µL EDTA (500 mM). The resulting signal, which remained stable for up to 5 hr after addition of stop reagent, was collected on a Molecular Devices SpectraMax M5 (absorbance at OD_340_)(Sunnyvale, CA).

To account for possible inhibition of the reporter enzyme in the primary coupled reaction, putative inhibitors were screened to assess their activity against a G6PDH coupled assay. Briefly, test and controls compounds were added to the wells of a 384 well assay plates as described above. Negative (vehicle) controls contained 1% DMSO, positive controls contained 100 mM PEP and IC_50_ controls contained 8.6 mM PEP (final well concentrations). A mixture containing glucose-6-phosphate (G6P, 0.6 mM) and NAD^+^ (1.8 mM) in a volume of 15 µL was then added to each assay plate well. The reaction was initiated by addition of 15 µL G6PDH (0.018 mUnits/mL) (for a final volume of 45 µl), incubated at RT for 1 hr, and then quenched with 5 µl of EDTA (500 mM). The change in absorbance at OD_340_ was monitored as above.

Additional specificity assays were performed using human HK 4 (human glucokinase, hGlk, GenBank accession no. BC001890) that was expressed from a cloned cDNA (OPEN Biosystems, Huntsville, AL) in pQE30. After sequencing, the plasmid was transformed into *E. coli* M15 (pREP) and cultures were grown to an OD_600_ of 0.9 in terrific broth and protein expression induced (3 hr, 37°C) with 1 mM IPTG followed by purification by nickel-affinity chromatography.

### Inhibition assays of lysate-derived TbHK

Parasite lysates from BSF parasites were prepared by incubation (5 min on ice) of 1.5×10^7^ cells in lysis buffer (0.1 M TEA, pH 7.4, and 0.1% Triton X-100) supplemented with 1 mM PMSF, 5 µg/ml leupeptin, and 100 µg/ml TLCK. In triplicate, cell equivalents (2×10^5^) were incubated with increasing concentrations of inhibitor for 15 minutes at RT prior to initiation of the coupled reaction. In short, the 200 µl reactions included 50 mM TEA, pH 7.4, 33 mM MgCl_2_, 20 mM glucose, 5.25 mM ATP, 0.75 mM NADP, and 0.1 units of G6PDH, with kinetic analyses performed using KaleidaGraph 4.1 (Synergy Software, Reading, PA).

### *T. brucei* viability assay

To determine the impact of TbHK1 inhibitors on cell growth, we seeded 5×10^3^ BSF parasites (cell line 90–13, a 427 strain) into 96-well clear-bottomed polystyrene plates in 200 µl HMI-9 supplemented with 10% fetal bovine serum and 10% Serum Plus (Sigma-Aldrich, St. Louis, MO) and grown in the presence of compound (2 µl) or equivalently diluted carrier for 3 days in 5% CO_2_ at 37°C. CellTiter Blue (Promega, Madison WI) was added (20 µl) and the plates incubated an additional 3 hr under standard culture conditions. Fluorescence emission at 585 nm was then measured after excitation at 546 nm in a GENios microtiter plate reader (Phenix Research Products, Hayward CA). DMSO solvent was maintained at or below 1%, with 1% causing a 16% reduction in cell number at the end of the three day assay.

Procyclic form (PF) parasites (29–13, a 427 strain, 5×10^4^/well) were grown in 96-well clear-bottomed polystyrene plates in 200 µl SDM-79 for 2 days (5% CO_2_, 25°C) and then CellTiter Blue (20 µl) added. Plates were then incubated for 1 hr under standard culture conditions. Fluorescence of samples was then characterized as above.

### Mammalian cell-line and *Leishmania* promastigote specificity assays

Cell-based specificity assays were performed as previously described [11]. Briefly, mammalian cell line and *Leishmania* promastigote assays were performed in final volumes of 25 µL using our previously described 384-well microtiter format [12]. All mammalian cell lines were cultured and maintained in complete growth medium preparations according to ATCC specifications (ATCC, Manassas, VA). *Leishmania* promastigotes were cultured as previously described [11]. A549 (1,000 cells/22 µL), IMR-90 (1,000 cells/22 µL), HeLa (1,000 cells/22 µL), MDA-MB-231 (3,000 cells/22 µL), *Leishmania* promastigotes (5,000 parasites/22 µL) were seeded into each well of 384-well microtiter plates and test and control compounds (3 µl) were added to individual wells. Vehicle and positive controls were 1% and 10% DMSO, respectively. For mammalian cells, assay plates were incubated for 44–46 h at 37°C in the presence of 5% CO_2_ and for the *Leishmania* promastigotes, assay plates were incubated for 44 h at 28°C with 5% CO_2_. Five µL of CellTiter Blue reagent was added to each assay plate well and incubated for 2–4 h at 37°C with 5% CO_2_. Data were captured on a Molecular Devices SpectraMax M5 (excitation A_560_; emission A_590_).

### HTS data analysis and statistical analysis

Primary HTS data analysis and subsequent compound IC_50_ calculations were performed using ActivityBase (IDBS, Guilford, UK) and Cytominer (University of Pittsburgh Drug Discovery Institute, Pittsburgh, PA). Structural similarity of the confirmed inhibitors was determined using Leadscope software (Columbus, OH). Additional visualization and statistical analysis were performed using GraphPad Prism software 5.0 and Spotfire (Somerville, MA). The PubChem database (http://PubChem.ncbi.nim.nih.gov) was used to verify if the confirmed TbHK1 small molecule inhibitors exhibited bioactivity in other assays.

### *In silico* ADME/toxicity analysis

Computational modeling tools were used to estimate the bioavailability, aqueous solubility, blood brain barrier potential, human intestinal absorption, the cytochrome P450 (*i.e.* CYP2D6) enzyme inhibition potential, mutagenicity, and hERG inhibition of the confirmed TbHK1 inhibitors. The bioavailability, aqueous solubility, and human intestinal absorption were estimated using the ADME Boxes v4.0 software (Pharma Algorithms, Toronto, Canada), while mutagenicity and hERG inhibition were estimated with TOX Boxes v2.9 software (Pharma Algorithms, Toronto, Canada). The CYP2D6 inhibition and blood brain barrier potential were predicted using Accord for Excel 6.2.2 (Accelrys, Inc, San Diego).

## Results

### Validation of optimized HTS assay conditions using the LOPAC set

**Figure 1:**
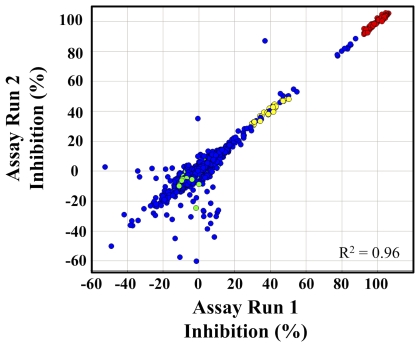
Validation of the HTS by LOPAC screening. Plot of percent inhibition for duplicate screen of the 1280 LOPAC compounds. LOPAC compounds (blue), minimum control, which should equal ∼100% inhibition of signal readout (red), IC_50_ control compounds (yellow), and maximum control compounds, which should equal ∼0% inhibition of signal readout (green), are indicated.

The TbHK1 coupled assay was optimized and validated for HTS by screening the LOPAC set. Compounds were assayed in duplicate at a single concentration (10 µM) and reproducibility between the duplicate screens is represented in Fig. 1 (R^2^ = 0.96). Average Z-factors were 0.69±0.02 for the two LOPAC assays demonstrating the robustness of the developed assay format [13]. Eighteen compounds inhibited TbHK1 enzymatic activity ≥40% at 10 µM including myricetin, a structural analog of quercetin, which was previously identified as a TbHK1 small molecule inhibitor (IC_50_ = ∼85 µM) (Lyda and Morris, unpublished). These data confirmed that our automated HTS assay conditions were robust and could be used to identify compounds that inhibited TbHK1 activity.

### Interrogation of 220,233 compounds for TbHK1 small molecule inhibitors

**Figure 2:**
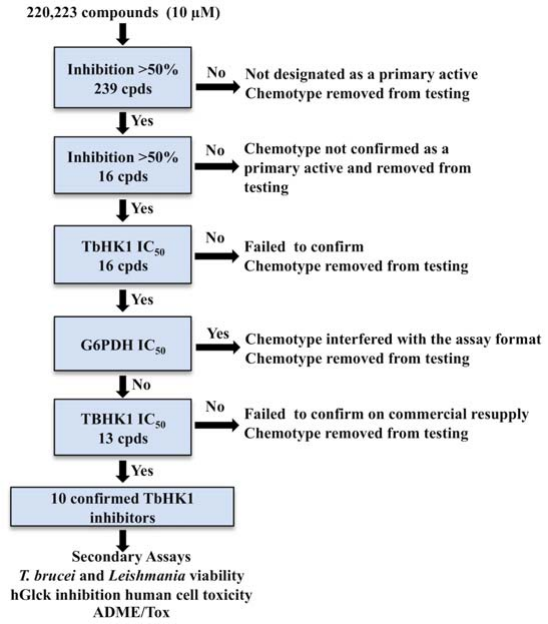
Scheme depicting HTS interrogation of a 220,233 small molecule library for TbHK1 inhibitors.

**Figure 3:**
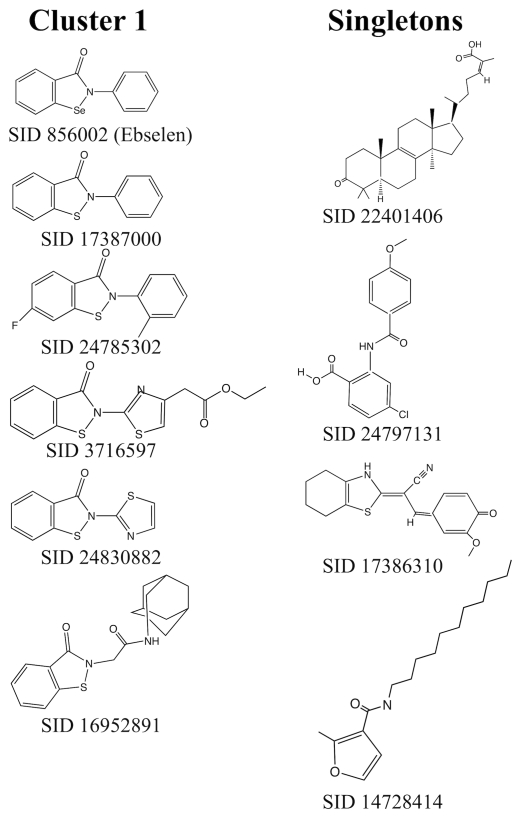
Structures of the cluster 1 and singleton HTS hits.

We next screened 220,223 compounds at a single concentration (10 µM) for small molecule inhibitors of TbHK1 (Fig. 2). The HTS assay performed robustly (average Z-factors of 0.80±0.1) and identified 239 compounds as primary actives (>50% inhibition at 10 µM), for an overall hit rate of 0.1%. The 239 active compounds were cherry-picked, and the initial inhibitory activity confirmed in the primary TbHK1 assay. Additionally, the compounds were tested against the reporter enzyme, G6PDH, to confirm that they did not interfere with the assay format. Following initial 20 point IC_50_ value determinations using cherrypicked compounds, compounds with IC_50_ values <50 µM were obtained from commercial sources. The activity of the 13 resupplied compounds was empirically determined to control for possible TbHK1 inhibitory effects associated with compound library degradation. Ten small molecules confirmed with TbHK1 IC_50_ values <50 µM while three compounds failed to inhibit TbHK1. Leadscope analysis of the 10 confirmed TbHK1 inhibitors classified six compounds into a cluster of structurally related compounds (cluster 1) while the remaining four compounds were classified as singletons (Table 1, Fig. 3). Ebselen (SID 856002) was the most potent compound in cluster 1 with an IC_50_ = 0.05±0.03 µM. For the majority of the compounds IC_50_ values either improved or remained similar to cherry-picked compounds with the exceptions of SID 17386310 and SID 14728414 (Table 1) which were 7.5 and 6.6-fold less potent, respectively, upon resupply (data not shown). Moreover, all ten novel TbHK1 inhibitors were 20-17,000-fold more potent than londiamine, a previously described TbHK1 inhibitor [4] and 2-1720-fold more potent than quercetin (Lyda and Morris, unpublished).

**Table 1:** HTS Cluster 1 and Singleton hits.

Leadscope Grouping	PubChem SID	PubChem Bioassay Activity^1^	IC_50_ (µM) Resupply	% Inhibition of hGlck (10 µM)	BSF EC_50_ (µM)	% PF Growth Inhibition (10 µM)	L. major EC_50_ (µM)
Cluster 1	856002 (Ebselen)	344/44/22	0.05±0.03	97.8±0.1	2.9±0.28	51±0.16	4.1±0.4
	17387000	236/38/17	2.0±0.5	6.7±9.4	0.030±0.067	48±0.15	1.9±0.2
	24785302	170/21/11	4.2±1.0	6.9±4.2	0.042±0.0028	47±0.15	1.9±0.2
	3716597	318/29/18	9.3±0.3	7.8±7.1	>10	27±0.080	>12.5
	24830882	171/12/7	16.9±0.1	88.8±4.9	0.83±0.20	8.6±0.030	>12.5
	16952891	214/24/11	2.6±0.2	44.9±9.9	0.30±0.079	47±0.15	>12.5
Singletons	22401406	184/5/2	2.3±0.3	0.0	>10	0.0	>12.5
	24797131	175/6/2	11.4±3.2	6.3±8.8	>10	0.0	>12.5
	17386310	211/10/4	33.6±10.2	70.3±3.6	0.038±0.0038	50±0.15	2.6±0.1
	14728414	216/2/1	41.7±3.0	1.9±7.0	>10	0.0	>12.5

1 As of 09/03/09. Number of bioassays in which the compound was tested/number in which the compound was active/number in which the compound has been confirmed as an inhibitor.

Additional experiments to assess the *in vitro* specificity included testing the compounds against hGlk. The activity against hGlck was varied, with the cluster 1 compounds yielding a spectrum of efficacy, from very low inhibition at 10 µM (for example, SID 17387000, with 6.7% inhibition, Table 1) to near complete inhibition by ebselen (97.8% inhibition). Singletons also demonstrated a spectrum of activity against hGlck with SID 22401406 and SID 14728414 having minimal impact on the enzyme while 17386310 was a more potent inhibitor (Table 1). Moreover, data mining of the PubChem database (http://pubchem.ncbi.nlm.nih.gov/) determined the frequency with which a compound was found to be active in other assays. In general, the cluster 1 compounds were active in other assays more frequently than the singletons, with SID 17387000 the most frequently active (identified in 7.2% of the 238 assays in which it was tested). Singletons, on the other hand, were less frequently active. For example, SID 14728414 was confirmed as an active in one of 216 assays (<1%) (Table 1).

### TbHK1 small molecule inhibitors are toxic to BSF parasites

TbHK1 has previously been shown to be an essential gene for BSF *T. brucei* [4], suggesting that inhibitors of the enzyme may be promising lead compounds for therapeutic development. To initially explore this possibility, we grew cultured BSF parasites in the presence of 10 µM compound and cell density monitored after 72 hr (Table 1). The ten resupplied compounds were tested in this assay, with EC_50_s determined for those that inhibited cell growth >50% at 10 µM (Table 1). Compounds in cluster 1 included two of the most potent anti-trypanosomal compounds, SID 17387000 and SID 24785302. Singletons were also toxic, with SID 17386310 being one of the most potent compounds tested to date. Four molecules, including a member of cluster 1 (SID 3716597) and three singletons (SID 22401406, SID 24797131, SID 14728414) inhibited TbHK1 but were not toxic to BSF at 10 µM. With the exception of ebselen, the resupplied anti-parasitic compounds exhibited EC_50_ values that were 10–1000 fold lower than the TbHK1 IC_50_ values. This discrepancy could result from other actions or concentration of the inhibitors in the glycosome, which is a small peroxisome-like organelle where TbHK1 is located. Additionally, we have found that RNAi of TbHK1, which reduces expression but does not necessarily ablate it, is toxic to BSF parasites, suggesting that modest inhibition of cellular TbHK1 activity could be lethal to the parasite [4].

To explore the likelihood of off-target whole parasite effects, we assessed the toxicity of the TbHK1 inhibitors against PF parasites. Unlike BSF parasites, PF parasites can utilize both amino acids and glucose for ATP production. This dynamic metabolism suggests that the PF parasites may be less sensitive to TbHK1 inhibitors. Indeed, at 10 µM most of the resupplied compounds had only a modest impact on PF parasite growth, inhibiting growth between 0–51% when compared to control cell lines. Compounds toxic to *T. brucei* were also assayed against a related kinetoplastid parasite, *Leishmania*. The *Leishmania* promastigotes were typically less sensitive to the resupplied compounds (with EC_50_s >12.5 µM), with the exception of the cluster 1 compounds ebselen, SID 17387000, SID 24785302 and the singleton, SID 17386310. These compounds had EC_50_ values against *Leishmania* between 1–5 µM in exponentially growing parasites (Table 1). Moreover, our identified TbHK1 inhibitors have minimal impact on human cell lines with EC_50_ values >12.5 µM, suggesting at least 400-fold greater toxicity toward parasites for the most potent *T. brucei* cytotoxic compounds (Table 1).

### *In silico* predictions for the identified TbHK1 inhibitors

To investigate the chemical similarity of our newly identified TbHK1 small molecule inhibitors to current treatments for African sleeping sickness as well as previously described TbHK1 inhibitors, we performed a similarity search using the Tanimoto coefficient. The data indicated that the TbHK1 inhibitors displayed low levels of similarity with all compounds examined with the highest similarity being between lonidamine and SID 16952891 (47%) (Table 2). Thus, these results demonstrate that the newly identified TbHK1 inhibitors are unique, either displaying no or very low similarity to known TbHK1 inhibitors and current therapies for African sleeping sickness.

**Table 2:** Comparison of structural similarities of HTS hits to licensed compounds used against HAT and to known TbHK1 inhibitors.

Compound	Proposed Mode of Antiparasitic Action^1^	TbHK1 Inhibitor (SID)	Similarity Coefficient^2^(%)
Pentamidine	Accumulation in the mitochondria, DNA binding	17386310	31
Suramin	Inhibition of glycolysis	24830882	38
		24797131	38
Melarsoprol	Inhibition of glycolysis Interaction with thiols	24830882	43
Eflornithine	Polyamine biosynthesis (via inhibition of ODC)	14728414	27
Lonidamine	TbHK1 inhibitor^3^	16952891	47
Quercetin	TbHK1 inhibitor^4^	24797131	38

1 Reviewed in [13].

2 Similarity coefficient was determined using the Tanimoto coefficient. Compounds with values greater than 80% are considered highly structurally similar.

3 [4].

4 Lyda and Morris, unpublished.

Additional *in silico* ADME-tox predictions indicated that all ten TbHK1 compounds had an extremely low probability for being either a hERG channel inhibitor or mutagenic (data not shown). Moreover, all compounds, except SID 3716597 were predicted to be moderately to highly bioavailable and nine of 10 compounds displayed medium to very high blood brain barrier (BBB) potential, with the majority of the cluster one compounds predicted to have high to very high BBB potential (Table 3). Six of 10 TbHK1 inhibitors were predicted to have no inhibitory activity on CYP2D6 enzyme with the exceptions being cluster one compounds SID 3716597 and SID 24785301 and singletons SID 14728414 and SID 24797131 (Table 3). The majority of the compounds had a predicted low aqueous solubility (Table 3), suggesting that if these compounds were to be used in future analogue development, they would need to be refined to improve their aqueous solubility. Thus, based on empirically derived data and *in silico* analyses, we focused on cluster 1 compounds for subsequent analyses.

**Table 3:** *In silico* ADME/toxicity analysis.

Leadscope Grouping	PubChem SID	Aqueous Solubility	Bioavailability	CYP2D6 inhibition	Blood brain barrier
Cluster 1	856002 (Ebselen)	Low	High	Non-inhibitor	High
	17387000	Low	High	Non-inhibitor	High
	24785302	Low	Moderate	Inhibitor	Very High
	3716597	Low	Low	Inhibitor	Medium
	24830882	Good	High	Non-inhibitor	High
	16952891	Low	Moderate	Non-inhibitor	Medium
Singletons	22401406	Extremely Low	Moderate	Non-inhibitor	Undefined
	24797131	Good	High	Inhibitor	Medium
	17386310	Good	Moderate	Non-inhibitor	Medium
	14728414	Low	Moderate	Inhibitor	High

### Further characterization of two structurally related cluster 1 TbHK1 small molecule inhibitors

**Figure 4:**
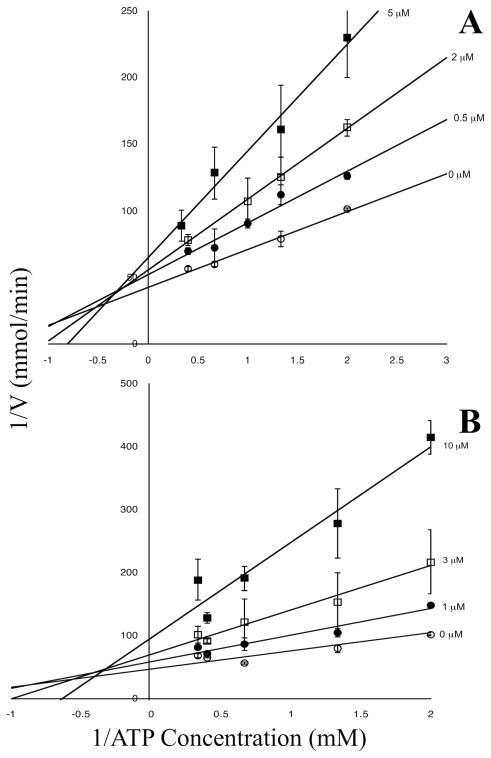
Ebselen and SID 17387000 are mixed inhibitors of TbHK1 with respect to ATP. Lineweaver-Burk plots of inhibition with ebselen (A.) or SID 17387000 (B.). Assays were performed as described for cell lystates (see Materials and Methods) with ATP concentrations varied.

**Figure 5:**
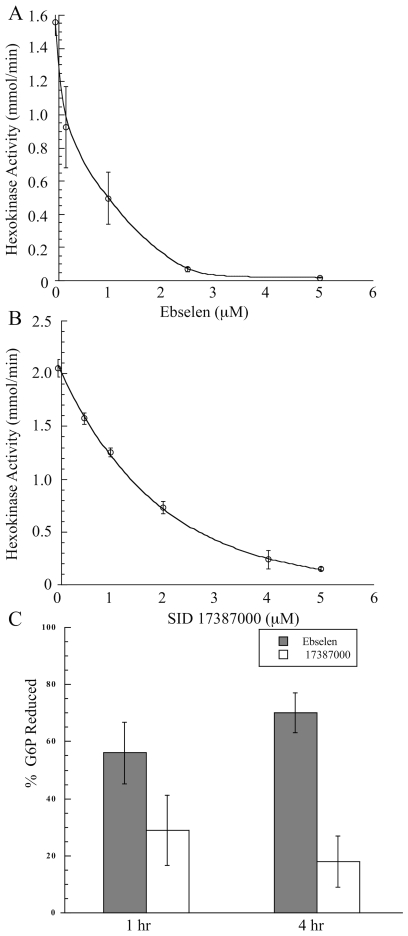
Ebselen and SID 17387000 inhibit TbHK activity from parasite cell lysate and cause a reduction in cellular G6P levels in BSF parasites. Increasing amounts of (A.) ebselen or (B.) SID 17387000 were incubated with 2×10^5^ BSF cell equivalents for 15 min at RT and HK assays were performed as described in the Materials and Methods. (C.) Growth in the presence of Ebselen or SID 17387000 causes a reduction in cellular G6P levels. BSF parasites (1×10^7^) were cultured for 1 or 4 hours in the presence of 30 µM or 1 µM (10-fold the EC_50_) ebselen or SID 17387000 followed by lysate preparation and comparison of G6P levels to an equivalent number of untreated parasites [14].

Ebselen, 2-phenyl-1,2-benzisoselenazol-3(2*H*)-one, was the most potent TbHK1 inhibitor (IC_50_  = 0.05±0.03 µM) identified in our studies while the structurally related inhibitor SID 17387000 (2-phenyl-1,2-benzisothiazol-3(2*H*)-one) was the next potent compound identified with an IC_50_  = 2.0±0.5 µM (Table 1). Analysis of the nature of TbHK1 inhibition revealed that both ebselen and SID 17387000 were mixed inhibitors with respect to ATP, with K_i_ values of 6.13 µM and 6.89 µM, respectively (Fig. 4). However, both ebselen and SID 17387000 exhibited comparable IC_50_ values against *T. brucei* lysate-derived TbHK1 enzymatic activity with IC_50_ values of 0.43±0.02 µM and 1.2±0.12 µM respectively (Fig. 5). Thus, while compound SID 17387000 was nearly as potent against lysate activity as the bacterially expressed protein, ebselen was significantly less potent against parasite lysate-derived TbHK1 activity (than the bacterially expressed protein). These results suggest that ebselen may be metabolized by cellular components or that lysate-derived TbHK1 may be associated with various cofactors that result in less potent IC_50_ values. Ebselen was also a potent inhibitor of hGlck (97.8% inhibition at 10 µM), while SID 17387000 had relatively little activity (6.7% inhibition) against the kinase (Table 1). Moreover, ebselen was ∼100-fold less active against BSF parasites (Table 1). Taken together, these data suggest that the subtle structural differences between the two compounds result in remarkable changes in their pharmacological behavior.

Both ebselen and SID 17387000 are potent trypanocides, with EC_50_s of 2.9±0.28 µM and 0.030±0.067 µM, respectively. To determine if indeed the toxicity to BSF parasites is related to inhibition of cellular TbHK1, we measured the impact of the compounds on cellular G6P levels after culturing the parasites in the presence of ebselen and SID 17387000 [14] (Fig. 5C). To reduce the likelihood that toxicity was impacting G6P levels non-specifically, we limited the incubation period to 1 and 4 hours, while employing high doses (10 times the EC_50_) of the compounds. Incubation with ebselen for either 1 or 4 hours led to a 56% or 70% reduction in G6P, while SID 17387000 was less effective, reducing G6P levels 29% and 18% after 1 and 4 hours, respectively (Fig. 5C). While these observations suggest a direct impact on TbHK activity, other off-target impacts could be ultimately responsible for toxicity.

## Discussion

There are currently four drugs approved for treatment of HAT. However, suramin and pentamidine, developed in 1921 and 1941, respectively, are not effective against the late stage of disease that occurs when the parasite crosses the blood-brain barrier. Melarsoprol, which was introduced in 1949, leads to fatal complications in 5–10% of patients receiving the drug [15]. The most recently developed drug, eflornithine, is only efficacious against *T. b. gambiense*, but is curative for both the early blood-borne infection and the late stage of disease with central nervous system involvement; delivery of eflornithine is difficult, as the compound must be administered intravenously four times a day for 14 days (delivering ∼360 g/patient).

A number of screens of chemical libraries have been undertaken to identify therapeutic leads against the African trypanosome. These include a phenotypic screen that interrogated a library of FDA-approved drugs for anti-trypanosomal activity [16], as well as screens developed to identify inhibitors of essential parasite enzymes. A screen for UDP-Glc 4′-epimerase inhibitors using a small natural products library [17] and a screen of a commercial 134,500 compound library for trypanothione reductase inhibitors [18] are two examples of target-based screens used to identify lead compounds for therapeutic development. In the last few years, TbHK inhibitors have been explored as potential anti-parasitic compounds. Previous efforts to identify TbHK inhibitors include the development of compounds based on models of the TbHK structure (predicted from homology studies of the yeast structure), and exploring the activity of HK inhibitors from other systems [4]. Here we have used a HTS of 220,223 compounds to identify new inhibitors of the parasite enzyme.

In our screens, we have identified several novel inhibitors of TbHK1. One compound, ebselen, was the most potent inhibitor from both the LOPAC validation screen and the HTS. Ebselen is a lipid-soluble seleno-organic compound that has been employed in clinical trials to assess its value in prevention of ischemic damage in brain hemorrhage and stroke [19], [20]. Ebselen inhibits lipid peroxidation through a glutathione peroxidase-like action [21], but may act through other mechanisms as well. Notably, a single oral dose (100 mg/kg) of ebselen yields serum values of 4–5 µM [22] and brain levels of the drug reach 21% of plasma levels [23], suggesting that the compound (or its derivatives) may be useful for both early and late stage sleeping sickness therapy development.

Ebselen likely has polypharmacological effects on BSF parasites, as the compound is known to inhibit a number of enzymes in addition to TbHK1, including the trypanosome UDP-Glc 4′-epimerase [17]. Ebselen, unlike other cluster 1 compounds, has an IC_50_ that is significantly lower than the EC_50_, suggesting its metabolism may be distinct from the sulfur-bearing compounds. Alternatively, a cellular “sink” could be interacting with ebselen, thereby lowering its effective concentration.

The remaining cluster 1 compounds have EC_50_s notably lower than their TbHK1 IC_50_s, suggesting possible actions on other cellular targets. Alternatively, differences between the two values could result from the concentration of the compound within the parasite (perhaps in the glycosome) or metabolism of the inhibitor to a more potent form. These hypotheses would seem worthy of further investigation.

An ideal therapeutic drug for African sleeping sickness would target the parasite (while perhaps enhancing host immune responses) and work at concentrations low enough to limit the severity of side effects. In the search for potential drug targets, we have focused on the trypanosome TbHK1, a protein that the parasite requires to make ATP and have identified compounds that may serve as leads in for the development of therapeutics in the continuing fight against the African trypanosome.
